# Disuse-Induced Muscle Fatigue: Facts and Assumptions

**DOI:** 10.3390/ijms25094984

**Published:** 2024-05-03

**Authors:** Xenia V. Sergeeva, Irina D. Lvova, Kristina A. Sharlo

**Affiliations:** Institute of Biomedical Problems, RAS, Khorosevskoye Shosse, 76a, 123007 Moscow, Russia; sergeeva_xenia@mail.ru (X.V.S.); irrrra1@yandex.ru (I.D.L.)

**Keywords:** unloading, disuse, fatigue

## Abstract

Skeletal muscle unloading occurs during a wide range of conditions, from space flight to bed rest. The unloaded muscle undergoes negative functional changes, which include increased fatigue. The mechanisms of unloading-induced fatigue are far from complete understanding and cannot be explained by muscle atrophy only. In this review, we summarize the data concerning unloading-induced fatigue in different muscles and different unloading models and provide several potential mechanisms of unloading-induced fatigue based on recent experimental data. The unloading-induced changes leading to increased fatigue include both neurobiological and intramuscular processes. The development of intramuscular fatigue seems to be mainly contributed by the transformation of soleus muscle fibers from a fatigue-resistant, “oxidative“ “slow” phenotype to a “fast” “glycolytic“ one. This process includes slow-to-fast fiber-type shift and mitochondrial density decline, as well as the disruption of activating signaling interconnections between slow-type myosin expression and mitochondrial biogenesis. A vast pool of relevant literature suggests that these events are triggered by the inactivation of muscle fibers in the early stages of muscle unloading, leading to the accumulation of high-energy phosphates and calcium ions in the myoplasm, as well as NO decrease. Disturbance of these secondary messengers leads to structural changes in muscles that, in turn, cause increased fatigue.

## 1. Introduction

The unloading (non-use or disuse) of skeletal muscles is characterized by a lack of contractile activity and mechanical load and can occur during gravitational unloading [1]. Muscle unloading is also observed in patients who are in long-term bed rest, after spinal cord traumas, during muscle immobilization, and during prolonged critical conditions (coma). Under such conditions, anti-gravity skeletal muscles do not need to support the weight and maintain the posture of the human or animal; locomotor muscles are also in a state of inactivity and are not performing the function of locomotion. After just a few days of unloading, functional changes in skeletal muscles are observed, in particular, a decrease in maximum contraction force, a decrease in muscle stiffness, and an increase in skeletal muscle fatigue, with the largest changes being in postural muscles, which humans need to maintain the vertical position of the body [2].

Muscle fatigue in a broad definition can be described as a muscle or muscle group’s inability to produce the given amount of static force or mechanical power during the preset time or an inability to perform the total amount of work under certain conditions [3,4]. Most often, the level of muscle fatigue is described as the ratio of the muscle contraction force at the end of a given period of time to the maximum muscle contraction force measured at the beginning of that period. Thus, the level of muscle fatigue does not directly depend on the absolute value of the maximum force of its contraction. However, fatigue depends on the mode (% of force from maximal contraction rate) of myofibres contraction [5].

At the moment, there is no precise understanding of the causes and mechanisms of increased fatigue during unloading [6], despite the fact that the mechanisms of the development of fatigue in skeletal muscles after physical exercise have been well studied [7]. Obviously, these mechanisms must be different, since both the structural and signaling changes that occur in muscle fibers during long-term inactivity are different from the changes that occur during exercise (although there are a number of similarities between these two conditions, which will be discussed below). It should also be noted that, although atrophy of muscle fibers occurs during muscle unloading, it has been shown in a number of cases that the prevention of the development of atrophy is not accompanied by a corresponding prevention of muscle fatigue [8], which implies that atrophy is not the only cause of fatigue related to muscle unloading.

## 2. Muscle Unloading-Induced Fatigue

The unloading-induced fatigue is observed in nearly all models of muscle unloading, although its presence is dependent on the time period and muscle type. Under conditions of a 6-day space flight, an increase in muscle fatigue in humans and animals has been shown, mainly in the muscles of the hind limbs. For example, after a 6-day spaceflight, rats’ soleus muscle fatigue, measured three hours after landing, was twice as high (34% of maximal force versus 64% at the end of the time period) as in controls [9]. In astronauts, both short-term (about 10 days) and long-term (more than 200 days) spaceflight resulted in increased fatigue in the knee extensor muscles [10]. However, a study published in 2010 found an increase in fatigue resistance in the knee extensor muscles of four astronauts who spent 181 days on the ISS, an effect that appears to be due to preventive measures taken during spaceflight [11]. 

The closest to space-flight effects on skeletal muscle is the on-ground model of microgravity—the “dry” immersion model [2]. This model implies the volunteer submerging in a warm water bath to the level of the neck, lying on a waterproof fabric [2]. Under conditions of “dry” immersion, there were no significant changes in the level of fatigue of the triceps surae [12], however, under conditions of a 7-day “dry” immersion, an increase in fatigue of both flexors and extensors of the knee joint was detected in a static test [13]. 

Bed rest is the most well-studied muscle disuse condition in terms of its effect on skeletal muscle fatigue. After 8 weeks of bed hypokinesia, fatigue of the knee extensor muscles doubled [14]. It has also been shown that after just five days of bed rest, the subjects’ ability to perform endurance exercises deteriorates [15]. However, after 21 days of bed rest, no decrease in resistance to fatigue of the plantar flexor muscles was found, despite a significant decrease in their volume [16].

Immobilization has a dual effect on skeletal muscle fatigue: in a number of experiments, fatigue of the elbow flexors decreased [17,18], while fatigue of the ankle flexors increased [19].

Under rat hind limb suspension, the level of fatigue of the soleus muscle increased significantly after 15 days [20], as well as after 7 days [21]. 

Thus, the muscles of the lower limbs, in particular the soleus muscle, are most susceptible to increased fatigue during muscle unloading in humans. And what could be the reasons for the unloading-induced fatigue?

The causes of muscle fatigue can be divided into central and peripheral influences. The group of central factors includes a decrease in the productivity of oxygen transport systems [22], which is determined by the state of the cardiorespiratory system. Hypokinesia of varying duration leads to a decrease in cardiac stroke volume and myocardial contractility alterations [23]; a decrease in blood plasma volume and an increase in hematocrit, which leads to a significant increase in blood viscosity [24]. It also causes a decrease in lung function, plasma volume, and red blood cell count resulting in lower arterial oxygen saturation [25] and development of hypoxemia. All of these changes can have serious consequences on multiple organ systems and may have a negative impact on exercise capacity [26] as a result of inadequate oxygen supply to the muscle tissue.

Central factors also include fatigue of the central and peripheral nervous system, leading to the inability of the central nervous system to provide sufficient activation of the motoneuron pools of contracting muscle groups, disruption of neuromuscular transmission, and conduction of excitation along the fiber. These changes may be caused by a decrease in corticospinal excitability [27], reciprocal inhibition provided by Ia afferents, increased negative feedback from type III and IV muscle afferents [28,29], and changes in the internal properties of the motor neuron [30]. In particular, these motor neuron property changes can result in variations in the level of persistent inward currents (PICs). The ability to generate persistent inward currents, caused by monoaminergic-dependent, voltage-gated calcium, and sodium channels, is an intrinsic property of the motor neuron that plays a key role in establishing the membrane potential in the subthreshold range, thereby regulating the process of depolarization. PICs increase the excitability of motor neurons and provide their self-sustaining firing [31,32,33].

A number of studies have shown that during prolonged muscular work, a decrease in PICs makes a significant contribution to the development of fatigue [31,32,33].

The effects of PICs are particularly important for the normal functioning of low-threshold slow motor units, which are most active during posture maintenance and walking [29]. It is worth emphasizing that under conditions of muscle unloading, it is the slow muscle fibers (which are components of the slow motor units) that undergo the most negative functional changes.

Interestingly, a recent study showed a hyperpolarizing shift in the resting potential after 21 days of suspension of mice’s hind limbs as a result of suppression of Na^+^ PICs in the motor neurons innervating the muscles of the hind limbs. This may be one of the possible mechanisms for the development of fatigue during muscle unloading. Although the exact mechanism of the decrease in transient Na^+^ current during muscle unloading is not yet known, alternative RNA splicing and translational repression of Na^+^ channels have in one study been suggested as a cause [34]. Another hypothesis is that the lack of ground reaction forces during unweighting affects the activation process of the intracellular domain of tropomyosin tyrosine kinase receptor B (TrkB) changing the probability of the Na^+^ channel opening [35]. Other neurophysiological studies have also shown decreased motor neuron excitability [36,37,38] and corticospinal excitability [39,40] during muscle immobilization and unloading. 

Another potential cause of muscle fatigue is neuromuscular junction (NMJ) alterations during muscle unloading. The role of neuromuscular junction disruption under muscle unloading in unloading-induced fatigue is still unexplored, although it can cause fatigue under various pathological conditions [41]. However, it was shown that the decreases in NMJ plate area occur in slow-type soleus as early as after 6–12 h of unloading [42]. 3 days of human dry immersion and 10 days of bed rest led to the NCAM-positive myofibre increase in the vastus lateralis muscle, which is known to be a marker of neuromuscular alterations [43,44]. At the later stages of rodent hind limb suspension, the changes in NMJ are more profound in fast-type muscles than in slow-type ones and are rather moderate [45]. 60 days of bed rest did not affect NJM secretome [46]. Several authors suggest that the alteration of NMJ during unloading followed by excitation-contraction alterations is the key reason for pre-atrophy early force loss during unloading [44]. However, the theory of support afferentation suggests that the loss of muscle tone due to lack of support stimuli is the key reason for the early unloading-induced force decline [47].

The muscular causes of fatigue include changes that directly occur in the muscle, such as deficiency of energy substrates, accumulation of metabolites, and ionic concentration changes. A muscle’s resistance to fatigue is determined by its oxidative potential, i.e., the muscle’s ability to receive and utilize oxygen. The oxidative potential of the muscle, in turn, consists of the level of blood flow to the muscle (in particular, the level of capillarization and the volume of blood circulating through the muscle capillaries), mitochondrial density, and mitochondrial activity, as well as the myosin phenotype of the muscle fiber. 

In addition to the fiber types, the resistance of a muscle to fatigue is determined by a number of factors that depend directly on the mode of activity of the muscle. These include changes in the level of energy substrates (ATP, ADP, CrP, glycogen), mechanical load (due to activation of mechanoreceptors), or changes in secondary messengers (calcium ions, nitric oxide, and reactive oxygen species (ROS)).

The early stages of muscle unloading are characterized by a rapid decrease in energy consumption by skeletal muscles and a subsequent increase in the ATP/ADP ratio and glycogen accumulation. There is also an accumulation of calcium ions in the myoplasm, production of reactive oxygen species (ROS), inactivation of mechanosensors, as well as a decrease in nitric oxide content [48,49,50]. In the soleus muscle, calcium accumulation in the myoplasm starts from the 3rd day of muscle unloading, and the calcium level, as well as the level of ROS, remains elevated at least during the first 14 days [51]. During active muscle work, a rapid increase in calcium levels is also observed, but then, during muscle relaxation and rest, the calcium level returns to normal values.

On days 1–3 of muscle unloading, accumulation of glycogen and ATP is also observed [49,52,53,54]. However, at later stages (day 14), the ATP level drops below the values typical for control animals [55]. In fact, in skeletal muscle (at least in the soleus muscle, which is the most studied under unloading conditions), parts of the signaling pathways are in a state characteristic of rest (decrease in the level of nitric oxide, accumulation of ATP and glycogen, and inactivation of mechanosensors) and other parts are in a state characteristic of active work (relatively high levels of calcium and ROS). It is important to note that during muscle unloading, protective mechanisms activated during contractile activity (such as the accumulation of NO and activation of protein synthesis) do not compensate for the negative impact of the accumulation of calcium ions and ROS, which may determine the differences between fatigue caused by muscle unloading and decreased fatigue resistance after prolonged physical activity. 

In each individual case, fatigue can be caused both by processes localized in the systems supporting the muscle performance and in the muscles themselves. It is worth noting that fatigue of various origins is associated primarily with the insufficient ability of the muscle groups involved in locomotion to carry out work of the required power and duration, and this, secondarily, leads to extreme activation of the body’s support systems. In accordance with this, we will next consider the main intramuscular factors of muscle fatigue in relation to conditions of muscle unloading.

## 3. Myosin Phenotype

Skeletal muscle consists of a mosaic of two fiber types that are called: “slow” and “fast”. The phenotype of the fiber itself determines which isoform of myosin heavy chains (MyCH) predominates in it. In total, there are four isoforms of MyHC, one “slow”—MyHC I(β) and three “fast”—MyHC IIa, IId/x, and IIb [56,57]. Due to the coordinated gene expression, each isoform of MyHC corresponds to certain isoforms of auxiliary proteins of muscle contraction, such as tropomyosin or troponin—all together these proteins provide the optimal mode of operation for this type of myosin. The fastest isoform of myosin, MyHC IIb, is not expressed in humans, despite the fact that its gene is present in the human genome [58]. The expression of a particular myosin isoform is determined by the pattern of fiber innervation [57]. Typically, with increasing intensity of exercise, the recruitment of motor units (MUs) occurs in accordance with the phenotype of the fibers included in this MU: low-threshold type I fibers are recruited first, and IId/x fibers in humans (or IIb in rodents) are recruited last, despite the fact that exceptions have been noted for a number of exercise modalities [59].

The expression of one or another myosin type determines the functional properties of the muscle fiber. J. J. Widrick et al. [60] compared gastrocnemius fibers of sedentary middle-aged men with runners. They found that in the sedentary group, there was no difference between fibers in peak force, whereas in peak tension (strength/fiber cross-sectional area, CSA) the fibers were arranged as follows: IId/x > IIa > Iβ; maximum shortening speed (Vmax): Id/x = IIa = Iβ; a/Pzero (where a is a constant with force dimensions and Pzero is peak isometric force): IId/x > IIa > Iβ; from which they concluded that type IId/x fibers produced twice the peak power of type IIa fibers, while type IIa fibers produced approximately five times the peak power of type Iβ fibers. Vmax and a/Pzero of type Iβ and IIa fibers did not differ between the groups of runners and sedentary people. Thus, the “fast” fibers, true to their name, contract faster than the “slow” fibers, and are capable of developing greater force. “Slow” fibers are characterized by slow contraction rates but increased resistance to fatigue [4]. 

The resistance of “slow” type fibers to fatigue, according to some authors, can be achieved precisely due to the lower rate of formation of the actomyosin complex. This hypothesis is supported by the fact that the use of an actomyosin interaction inhibitor in type II fibers increases their resistance to fatigue [61,62]. Thermodynamic efficiency (mechanical power per rate of energy release) of fast and slow fibers is the same, as well as the tension developed during a single actomyosin interaction, in contrast to the almost fourfold difference in the maximum mechanical power output and the rate of ATP hy-drolysis. In other words, the difference between myosin isoforms is not the proportion of ATP energy converted into mechanical work, but the rate at which energy conversion occurs [63]. Since the mitochondrial capacity in slow fibers is balanced with the ATPase activity of myosin, these fibers can resist fatigue for a long time, contracting under conditions of smooth tetanus. CrP reserves are retained for a longer time (taking into account the constant resynthesis of ATP during oxidative phosphorylation), and glycolysis products accumulate to a lesser extent. Moreover, slow fibers can use lactate from fast-type fibers as a fuel for mitochondria.

“Slow” type fibers contain a large number of mitochondria, myoglobin, and oxidative enzymes. The type of metabolism in such fibers is predominantly oxidative. Type IIa fibers have an intermediate position between the extreme types (I and IIb) and also contain a high number of mitochondria. These fibers are less fatigue-resistant than slow type fibers, but much more fatigue-resistant than “fast” fibers, and also have a mixed energy metabolism [64]. Fast-twitch fibers exhibit high contraction speed and force but also fatigue easily. They contain a relatively small number of mitochondria, myoglobin, and oxidative enzymes, which is due to the fact that the predominant type of metabolism in “fast” fibers is anaerobic oxidation of glucose (glycolysis). In this regard, the main energy substrates for fast-type fibers are glycogen and CrP reserves [57]. 

It is well known that conditions of muscle unloading are accompanied by a decrease in the expression of the “slow” isoform of MyHC, while the expression of the “fast” isoforms increases significantly, which leads to the transformation of the myosin phenotype [65,66,67]. Space flight, “dry” immersion and bed rest lead to the slow-to-fast fiber type shift [66,68,69,70,71]. It was shown that after 24 h of rodent hind limb suspension, the expression of MyHC Iβ mRNA in the soleus muscle decreases [53]. After 7 days or more of rodent hind limb suspension, the expression of the slow isoform of MyHC is significantly reduced at the mRNA level and also at the protein level [48,67,72,73,74]. The expression of mRNA of the “fast” oxidative isoform of MyHC IIa decreases from day 3 of rodent hind limb suspension, remaining at this level until day 7, then by day 14 it returns to the level of control values [67,75]. The expression of mRNA of the “fast” isoforms of MyHC IIb and IId/x increases dramatically from the first day, reaching a maximum by day 14, and remains elevated for at least 28 days of muscle unloading [67,73]. It is obvious that the transformation of the myosin phenotype should contribute to a decrease in muscle resistance to fatigue under conditions of muscle unloading. It should also be noted that in a number of experiments, under conditions of space flight and “dry” immersion, it was shown that atrophy of “slow” type fibers is more pronounced than atrophy of “fast” fibers, which can also contribute to an increase in muscle fatigue [2].

It is worth highlighting the signaling interaction between the regulation of the “slow” oxidative phenotype and mitochondrial biogenesis. Actomyosin interaction is the main consumer of energy in skeletal muscle during muscle contraction. Accordingly, it would be logical to assume that the parameters of energy consumption in skeletal muscle (i.e., the speed and force of contraction, determined, among other things, by the myosin phenotype) should correspond to the parameters of energy generation (i.e., type of fiber metabolism).

Indeed, a number of molecular regulators that ensure the expression of the “slow” isoform of myosin heavy chains also activates mitochondrial biogenesis, thereby enabling the functioning of the muscle fiber as an integrated system. In particular, under conditions of muscle unloading, AMP-activated protein kinase (AMPK) is a positive regulator of the expression of both the slow isoform of MyHC and PGC1α [53,54], and GSK-3beta is a negative regulator for both of them [72]. Under unloading, the accumulation of myoplasmic calcium appears to also be a negative regulator for both the slow myosin isoform and mitochondrial density. In particular, pharmacological activation of SERCA led to the prevention of slow fiber type depletion, as well as to the prevention of a decrease in mitochondrial density. In particular, pharmacological activation of SERCA led to the prevention of slow fiber type depletion, as well as to the prevention of a decrease in mitochondrial density. Moreover, while the mechanisms of the effect of excess myoplasmic calcium on mitochondrial density are still unclear, the reasons why preventing the accumulation of calcium prevents a decrease in the level of expression of slow-type myosin are apparently associated with the activation of the calcineurin-NFAT signaling pathway. NFATc1 is known to be activated by calcium-dependent phosphatase calcineurin [76]. However, under muscle unloading NFAT nuclear content and transcriptional activity is compromised despite the calcium level increase [77,78]. Recent articles suggest that activation of two NFAT-targeting protein kinases, MAP-kinase p38, and GSK-3beta, block NFATc1 during unloading and their inhibition activates NFAT signaling [72,79]. MAP-kinase p38, in turn, can be activated by CaMK II and ROS accumulation [80]. Administration of the SERCA activator during 7-day hind limb suspension prevented a decrease in the content of NFATc1 in myonuclei, and also led to a decrease in the level of phosphorylation of p38 MAP kinase, potentially due to CaMK II inactivation and ROS decrease [81]. So, under unloading conditions, the effect of SERCA activator administration on the downregulation of CaMK II/p38 seems to have a stronger activating effect on NFATc1 than its potential inhibition of calcineurin.

In addition to sharing the same upstream regulators with mitochondria, the slow isoform of MyHC can lead to activation of the expression of PGC1α due to the expression of microRNA, and PGC1α, which in turn, is able to activate the expression of the slow isoform of MyHC both directly and by increasing mitochondrial density and activation of mitokine expression. The work of J. Lin et al. [82] showed that PGC-1α, together with Mef2 proteins, activates transcription and serves as a target for calcineurin signaling, which is involved in the expression of slow fiber genes. And, the expression of the genes of the “slow” isoform of MyHC is accompanied by the expression of microRNA-208 and microRNA-499, which are capable of triggering the expression of PGC-1α [83,84]. This, for example, was shown in the work of Jing Liu et al. [85], where transgenic mice with overexpression of microRNA-499 were used. In particular, mi-croRNA-499 directly inhibits Fnip1, which is a negative regulator of AMPK. Reyes et al. [86] showed that in mice with Fnip1 knockout, the content of type I fibers is increased, as well as myoglobin, slow myosin gene expression, oxidative enzymes, the density and number of mitochondria, AMPK activity, and PGC-1α expression. The fibers of the knockout animals also had higher oxidative capacity and fatigue resistance. In response to the hind limb suspension of rats and in “dry” immersion in humans, microRNA-499 content decreases in the soleus muscle [84,87]. It is possible that this decrease contributes to the deregulation of both the expression of the slow myosin isoform and mitochondrial biogenesis. Also, mitokines—recently discovered regulators—can activate the expression of the “slow” isoform of MyHC as well as the biogenesis of mitochondria. Mitokines are short peptides produced by mitochondria during their active work [88]. The mitokine MOTS-c is able to inhibit the folate cycle, which in turn leads to a decrease in de novo purine synthesis, accumulation of AICAR (5-aminoimidazole-4-carboxamide ribonucleotide) and activation of AMPK. AMPK, like MOTS-c, partially mediates the activation of glycolysis, the pentose phosphate pathway, and fatty acid oxidation and promotes muscle endurance [89]. By the 7th day of rat hind limb suspension, both the expression of the MOTS-c precursor mRNA and MOTS-c content in the myoplasm decrease and with pharmacological prevention of the decrease in mitochondrial density (due to the administration of beta-guanidine propionic acid or nifedipine), its content in the myoplasm corresponds to values in the control group [90,91]. Our laboratory has shown that MOTS-c administration following 7-day rat hind limb suspension prevents the reduction in fatigue resistance of the soleus muscle (unpublished data), suggesting a role for MOTS-c reduction in the development of muscle fatigue induced by muscle unloading. 

Thus, during muscle unloading, both upstream activators and blockers of the expression of the slow isoform of MyHC and the level of mitochondrial density are deregulated. The disruption of horizontal signaling pathways between the expression of the slow isoform of MyHC and mitochondrial biogenesis also occurs which leads to the disruption of feedback loops that support slow, oxidative, and fatigue-resistant muscle fiber phenotype.

## 4. Oxidative Potential

Oxidative potential is the ability of skeletal muscle to consume oxygen. Factors influencing muscle oxidative potential include the activity of oxidative enzymes, fiber phenotype (predominance of one or another myosin isoform, mitochondrial density, number and activity of oxidative and glycolytic enzymes), and oxygen availability [92]. Oxygen availability is determined by the density of capillaries in skeletal muscle and the blood flow within them. Capillary density in muscle is a key factor influencing the diffusion of oxygen into mitochondria within the fiber [92]. The density and number of capillaries per fiber in the vastus lateralis muscle have been shown to decrease after several weeks of unilateral leg suspension, bed rest, and immobilization [77,93,94]. Under rat hind limb suspension, a decrease in capillarization was shown in the postural soleus muscle, which contains mostly “slow” type fibers, while in the “fast” plantaris muscle the level of capillarization remained at the control level [95]. In addition to a decrease in the level of capillarization after 21 days of rat hind limb suspension, a decrease in blood flow velocity was observed in the soleus and gastrocnemius muscles [96]. A decrease in blood flow velocity was also observed during bed rest in the human lower limb muscles [97]. However, it is worth noting that the use of vasodilators during unloading did not prevent the transformation of the myosin phenotype and muscle fiber atrophy in rats [98], so vasodilation alone does not help prevent the disuse-induced negative effects. It can be assumed that the lack of negative consequences of vasoconstriction during unloading is caused by the reduced energy consumption of unloaded muscles, and the decrease in blood flow velocity is not the cause of the negative changes caused by muscle unloading. Moreover, the use of artificial limitation of the level of blood flow during bed rest in humans prevents a number of negative changes caused by muscle unloading, including a decrease in the force of muscle contractions [99], apparently due to the enhancement of the expression of capillarization activators. 

Mitochondrial density and mitochondrial enzyme activity play a critical role in muscle resistance to fatigue, especially during endurance exercise. It has been shown that in human muscles, a decrease in motor activity is accompanied by a decrease in mitochondrial density and the maximum rate of mitochondrial respiration, which negatively affects the maximum rate of fat and carbohydrate oxidation and reduces aerobic capacity [100,101]. With long periods of bed rest (from 8 to 65 days), a decrease in the activity of a number of tricarboxylic acid cycle enzymes and oxidative phosphorylation has been shown in the vastus lateralis muscle [102,103], i.e., the oxidative capacity of skeletal muscles decreases rapidly and then remains at a low level. With long periods of bed rest the expression of many genes encoding mitochondrial proteins (tricarboxylic acid cycle (TCA) and beta-oxidation enzymes, electron transport chain proteins, and fatty acid transporters) also decreases [104,105,106,107,108,109,110]. After 6 and 35 days of bed rest the content of TCA cycle enzymes and oxidative phosphorylation enzymes in the vastus lateralis muscle decreases [111]. In experiments with 2-day leg immobilization, the expression of several genes, encoding mitochondrial proteins, is also reduced in the vastus lateralis muscle [104]. 

In a 3-day “dry” immersion experiment, it was shown that the intrinsic respiratory capacity of mitochondria decreases, which may be due to the disruption of the activity of respiratory complexes [112]. Under 6-day “dry” immersion, no decrease in mitochondrial density was detected, however, at the level of mRNA expression, there were decreases in the parameters of mitochondrial biogenesis, components of the respiratory chain, as well as mRNA expression of regulators of mitochondrial fusion and fission [87]. 

A decrease in the volumetric density of mitochondria was found in the subsarcolemmal region after a 14-day space flight in the soleus muscles of rhesus macaques [113]. In humans, a significant decrease in the mitochondrial density in the subsarcolemmal zone was observed after 60 days of bed rest, and after 120 days a decrease was also found in the central zone of the fibers [114,115]. Five-week rat hind limb suspension leads to a decrease in the volumetric density of subsarcolemmal mitochondria and an increase in the volumetric density of intermyofibrillar mitochondria, with overall preservation of the total volumetric density of mitochondria [116]. A total of 24 h of rat hind limb suspension causes a decrease in the mRNA of the regulators of mitochondrial biogenesis and fusion [54]; by the 7th day, a decrease in the level of mitochondrial DNA and markers of mitochondrial density is also observed [21,81]. The same situation persists on the 14th day [117].

The reasons for the decrease in mitochondrial density during the initial stages of muscle unloading include the accumulation of ATP, accompanied by inactivation of AMPK. Indeed, preventing the accumulation of ATP on the 1st day of muscle unloading leads to the prevention of a decrease in mRNA of mitochondrial biogenesis parameters. On the 7th day, it helps to maintain mitochondrial DNA level and prevent the development of fatigue [54,91]. At the same time, it is known that during long periods of unloading, AMPK activation occurs [118]. However, the level of mitochondrial density and the activity of some mitochondrial enzymes remain reduced under these conditions [117]. Thus, AMPK activity in the late stages of muscle unloading, in contrast to the early stages, is apparently insufficient to maintain mitochondrial density.

A number of experimental data indicate that excessive intracellular calcium accumulation may contribute to a decrease in mitochondrial density under muscle unloading. The administration of both the L-type calcium channel blocker nifedipine and the SERCA activator prevented a decrease in the content of mitochondrial DNA and the mitochondrial density marker protein TOM20 [81,90]. They both also prevented the decrease in the expression of mRNA of a number of mitochondrial biogenesis regulators, which was quite unexpected, since it is widely known that the accumulation, but not a decrease, in calcium levels leads to activation of mitochondrial biogenesis [119]. The mechanisms that determine the beneficial effect of pharmacological reduction of calcium levels on mitochondrial density during muscle unloading have yet to be studied.

Nitric oxide (NO) content decline was observed in the soleus muscle on the 7th and 14th days of rat hind limb suspension [48,72]. The role of NO decline in the unloading-indued drop in mitochondrial density is poorly studied. It was shown that the administration of the NO donor L-arginine during 7 days of unloading prevented a decrease in the PGC1α content, while the combined administration of L-arginine and the NO synthase inhibitor L-NAME abolished this effect [72]. It has also been shown that mechanical stimulation of the feet support zones during 7-day rat hind limb suspension leads to both the prevention of increased fatigue and mitochondrial DNA copy number decline in the soleus muscle [21]. It has previously been shown that mechanical stimulation of the feet support zones prevents NO content downregulation in the myoplasm of soleus muscle fibers [120], but it is unknown whether NO is a regulator of the levels of fatigue under these conditions. However, it has been shown that the nitric oxide target kinase GSK-3beta, which is activated under conditions of NO deficiency during unloading [72], contributes to the decrease in the content of PGC1α, a number of mitochondrial proteins and mitochondrial DNA in the soleus muscle under hind limb suspension [117,121,122].

The epigenetic mechanisms of inactivation of mitochondrial biogenesis tend to also be involved in the unloading-induced downregulation of mitochondrial biogenesis and function. An increase in the level of methylation of CpG islands in the promoter region of the PGC1 gene was shown after 7-day rat hind limb suspension and after 6-day “dry” immersion in the soleus muscle. The same changes were reported after bed rest in the vastus lateralis muscle [21,87,106]. It is possible that the methylation of the PGC1α promoter also contributes to the decrease in mitochondrial density under conditions of muscle unloading of skeletal muscles.

Among the reasons leading to the unloading-induced decrease in mitochondrial density, one that should be noted is the transformation of the myosin phenotype. As already mentioned, the expression of the slow MyHC isoform and the biogenesis of mitochondria have a number of mutually activating signaling mechanisms (micro-RNA, PGC1α, mitokines), as well as a number of upstream regulators (AMPK, GSK-3β, calcium ions). It is possible that with pharmacological or physiotherapeutic (mechanical stimulation, electrical stimulation) restoration of the normal myosin phenotype, the functioning of mitochondria will also return to normal; in turn, any method of restoring the normal level of mitochondrial biogenesis and functions may have a positive effect on the myosin phenotype of the muscle.

## 5. Calcium

The calcium signal is essential for the initiation of muscle contraction. The most popular and simple way to express the response of the myofibrillar apparatus to changes in calcium concentration is the relation between the force developed by the muscle or muscle fiber and the free calcium concentration pCa (-log [Ca^2+^]). The pCa50 (half-maximal) value is an indicator of the affinity of myofibrils to calcium. Myofibrillar calcium sensitivity is usually studied in fibers by measuring steady-state isometric force in solutions of varying calcium concentrations [123], but force–pCa curves have also been obtained in intact muscle fibers [124]. A curve shifted to the left indicates increased sensitivity to calcium (for the same signal strength, greater force will be developed), while a curve shifted to the right indicates a decrease in sensitivity to calcium (and increased fatigue, since for the same signal strength, less force will be developed).

Rat hind limb suspension experiments showed that after 1, 2, and 3 weeks of suspension, force-pCa and stiffness-pCa curves for the soleus muscles were shifted to the right [125]. 17 days of bed hypokinesia also resulted in decreased calcium sensitivity of human soleus muscle fibers [126]. The same effect was observed after a 7-day “dry” immersion [2]. A decrease in sensitivity to calcium (a significant increase in pCa50) was also obtained after a 45-day space flight for type I (but not type II) fibers [127]. 

Calcium sensitivity, as measured by the pCa50 parameter of the calcium-force curve, can be influenced by a number of factors, such as temperature, pH, sarcomere length, ionic strength, myofilament spacing, Pi concentration, ATP and ADP content, and post-translational modification of myofibrillar proteins (for example, phosphorylation of myosin regulatory light chains). Slow-twitch fibers have a lower activation threshold and a less steep calcium curve slope than fast-twitch fibers, which contributes to differences in contractile force and velocity between different fiber types [128]. What factors or combinations of factors determine the shift in the calcium-force curve under conditions of muscle unloading remains unclear.

In addition to its role in muscle contraction, calcium is also a signaling molecule that may contribute to the rate of muscle fatigue development. Calcium ions accumulate in the myoplasm as early as after the second day of soleus muscle unloading [51,129]. Calcium also transiently accumulates in the muscle during physical activity, while during unloading, a relatively small in amplitude, but long-lasting increase in the content of calcium ions is observed. This is similar to what is observed with aging, as well as in various pathological conditions, such as Duchenne muscular dystrophy [130]. Long-term accumulation of calcium ions in the myoplasm can lead to the accumulation of ROS [131]. It has been shown that in some cases, for example in aging and in the diaphragm during mechanical ventilation, ROS can lead to the oxidation and destabilization of ryanodine receptors (RyRs) and their spontaneous opening [132,133,134]. Chronically opened RyRs cause further accumulation of calcium ions and ROS in muscle fibers. It further leads to the depletion of calcium stores in the sarcoplasmic reticulum, decreasing the force of muscle contraction and reducing resistance to fatigue [132]. It is possible that this mechanism may contribute to an increase in muscle fatigue under conditions of muscle unloading.

Elevated levels of intracellular calcium can also cause disruption of communication between dihydropyridine receptors (DHPR) and RyRs in the SR. It was found that the junctophilin-1 protein, which connects these two channels, undergoes calcium-dependent proteolysis caused by autolytic activation of endogenous μ-calpain. The authors suggest that the degradation of junktophilin-1 may contribute to increased skeletal muscle fatigue [135]. Our laboratory found a decrease in the junctophilin content in the total protein fraction of the soleus muscle after 7 days of muscle unloading (unpublished data).

In 2023, our laboratory showed that the administration of the L-type calcium channel blocker nifedipine, as well as the administration of the pharmacological activator SERCA, during 7-day rat hind limb suspension, prevented an increase in soleus muscle fatigue [81,90]. Also, as mentioned above, in this experiment, in the nifedipine-treated group, a decrease in a number of markers of mitochondrial density and mitochondrial biogenesis was prevented. In addition, it was shown that the administration of nifedipine during 14 days of unloading prevented the slow-to-fast fiber type shift [136]. Thus, excess calcium accumulation under muscle unloading can affect fatigue both directly and through the myosin phenotype and mitochondria.

## 6. Metabolites

**Figure 1:**
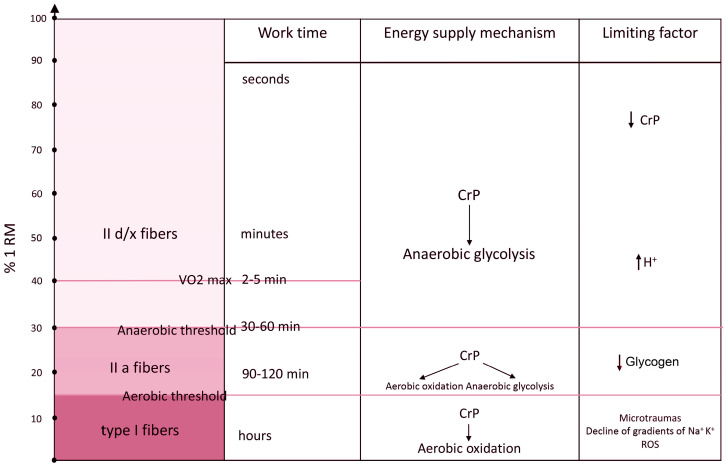
Relation of the maximum possible work time, energy supply mechanisms, and factors limiting muscle work to the intensity of muscle work. See text for explanations. % 1RM—percentage of power from one-repetition maximum, CrP—creatine phosphate.

In order to correctly describe the role of the accumulation and expenditure of energy metabolites in the development of muscle fatigue, it is necessary to determine the range of loads in consideration. During exercise, the contribution of various metabolic pathways providing energy for muscle contractions is determined by the relative intensity and absolute power output of the exercise (Figure 1). The relative intensity determines the type of fibers involved, and the power output determines the rate of ATP utilization and the mechanism of energy supply.

Thus, during low-intensity contractions that can be sustained for more than 120 min, due to low external resistance, low-threshold slow muscle fibers are recruited [5]. With the beginning of the contractions, Ca^2+^ and ADP ions appear in the sarcoplasm, which activates creatine phosphokinase, shifting the rephosphorylation reaction of CrP and ADP to the right, which allows ATP to be resynthesized in a few milliseconds [137]. The accumulation of allosteric regulators such as Pi, AMP, and ADP, NH_4_ activates the enzymes phosphorylase and phosphofructokinase, and the appearance of free Cr immediately triggers the creatine phosphate shuttle and the rapid activation of mitochondrial respiration, the substrates for which are pyruvate and free fatty acids [138,139]. 

Since the ATPase activity of myosin in slow fibers corresponds to the rate of energy production during mitochondrial respiration, the contraction power of these fibers should not alter significantly when changing from a powerful energy source (CrP reaction) to a less powerful one (oxidative phosphorylation). In addition, the concentration of CrP remains at a relatively constant level (about 70–80% of the initial concentration [140]), due to the constant resynthesis as a result of the functioning of the creatine phosphate shuttle.

As the relative exercise intensity increases, the activating influence of the central nervous system also increases, and this leads to the involvement of new MU. Heart rate, oxygen consumption, and pulmonary ventilation gradually increase. When the power output reaches a certain value, a moment comes when all slow muscle fibers (Type I) are involved in the work and intermediate muscle fibers (Type IIa) begin to be recruited. This point of transition from purely aerobic to aerobic/anaerobic energy metabolism is called the aerobic threshold (AT). As a criterion for the onset of anaerobic glycolysis, a fixed concentration of blood lactate is used—2 mmol/L—which is the upper limit of normal for the concentration of lactate at rest [92]. Most of the energy at a given level of exercise intensity is produced through the oxidation of plasmatic free fatty acids [141], which is likely due to lower accumulation of metabolic regulators such as ADP, AMP, and Pi than that generated by more intense exercise loads. It has also been hypothesized that increased availability of free fatty acids in plasma may reduce the utilization of carbohydrates as substrates for oxidation by suppressing the activity of the pyruvate dehydrogenase complex (PDC). This is affected by the increase in the acetyl-CoA/CoA ratio in mitochondria, as well as by the inhibitory effect of high citrate concentrations on phosphofructokinase activity [142]. 

Fatigue during prolonged low-level muscle activity may be caused by changes in supraspinal mechanisms that regulate neural excitation of working muscle [143,144].

At the spinal level, decreased excitability of motor neurons may be the result of sensitization of small afferent fibers (groups III–IV) due to proinflammatory mediators produced during prolonged physical activity [145,146,147]. 

At the muscle fiber level, muscle damage may be one of the causes of fatigue [144,147,148,149]. It was shown that long-distance running (100 km) leads to Ca^2+^ accumulation in skeletal muscle. This is accompanied by cell damage, which was assessed by measuring the leakage of muscle-specific enzymes [150].

Another possible factor limiting performance during prolonged work is the significant reduction in transmembrane gradients for Na^+^ and K^+^ in skeletal muscle fibers [151]. There is a possibility that if the trans-sarcolemmal K^+^ gradient (or Na^+^ gradient) is reduced, this may lead to decreased muscle function due to lower muscle fiber excitability [150].

A further increase in the intensity requires the recruitment of higher threshold IIa fibers, where, relative to CrP expenditure, the processes of aerobic oxidation of fatty acids and carbohydrates are activated since this type of fiber is also characterized by high activity of mitochondrial enzymes. The rate of glycolysis and the subsequent entry of pyruvate into mitochondria for oxidation corresponds to the need for ATP. This is known as aerobic glycolysis. Mitochondria in untrained skeletal muscle can regulate the rate of pyruvate and cytosolic electron pair uptake and oxidation across at least a 150-fold dynamic range from rest to moderate-intensity exercise (calf extension exercise), but the catalytic potential of lactate dehydrogenase (LDH) is still approximately 340-fold higher [152]. In other words, the LDH response maintains a near-equilibrium state over a very wide functional range of muscle activity. However, anaerobic glycolysis is also active, which is accompanied by the release of lactate and H^+^ ions. Due to their small size, they easily diffuse into the blood and neighboring slow fibers [153], but this does not significantly change the pH of the muscles. Half of the energy demand in this power zone is supplied by carbohydrates, mainly from muscle glycogen with about fifth of this being supplied by the liver. The second half of the energy demand is provided by intramuscular triglycerides and free fatty acids in the blood plasma [141]. In absolute values, the rate of fat oxidation reaches its maximum, which has been reported to correspond to ~60–65% of VO_2_max [137]. Moreover, the higher the power output the greater the contribution of carbohydrates to the overall energy metabolism [154,155].

In this case, fatigue-induced tension reduction is caused by the depletion of intramuscular carbohydrate reserves. The decrease in oxidative resynthesis of ATP, the accumulation of ADP and Pi in type I fibers during the first hour of muscle work, and in type II fibers towards the end of the workout is in proportion to the glycogen stores depletion in type I fibers [156,157,158].

Recruitment of an increasing number of fast IIa, and then IId/x fibers, as a result of increasing external loading, is accompanied by an increase in the rate of ATP hydrolysis. In these fibers, as CrP depletes, glycolysis and oxidative phosphorylation are activated. But, the ATP turnover can no longer be uniquely provided by mitochondrial respiration. As a result, the production of pyruvate, exceeding the capacity of mitochondria to absorb it, leads to the accumulation of lactate and H^+^ protons in the sarcoplasm of the muscle fiber. At this point, the substrates for oxidation in all active muscle fibers are predominantly carbohydrates and lactate produced by glycolytic fibers [159]. 

It should be noted that there is no instantaneous decrease in cellular pH since the fiber has a set of buffer systems, the main of which are: HPO_4_^2−^ ions, CrP hydrolysis, lactate production [160], and most importantly, the mitochondria of neighboring oxidative fibers, where NADH oxidation, not associated with ATP resynthesis, has a buffering effect. This dynamic equilibrium between the rate of formation of the end products of glycolysis and the rate of their utilization is called the anaerobic threshold (AT) [92]. A clear relationship has been identified between the power output (or rate of oxygen consumption), at which the AT occurs, and the level of aerobic performance, since the AT reflects the maximum oxidative potential of the fibers and their buffer capacity, or, in other words, the mitochondrial mass of the muscle fibers participating in the exercise. The AT corresponds to the maximum power a person can maintain for several tens of minutes without progressive fatigue.

Above the AT, workloads are carried out due to the involvement of an additional number of glycolytic fibers IId/x, the degree of participation of which is determined by the amount the threshold power is exceeded. It should be noted that the mechanical power output of such loading intensity is inversely proportional to the length of time muscle work is performed. Oxygen consumption gradually continues to increase, but this is no longer associated with oxygen consumption by the working muscles. Respiratory muscles and cardiac muscles are now contributing to extra oxygen utilization since they also increase their activity. As muscle fibers reduce their mechanical productivity as a result of glycogen depletion or an increase in the loading, the motor zone of the cerebral cortex increases its activating influence on the alpha-motoneuron pool of the muscles, which leads to the recruitment of additional high-threshold glycolytic motor units and an increase in the firing rate of those already working. As a result of the recruitment of a significant number of glycolytic fibers and due to the difference between the number of metabolites produced from anaerobic glycolysis and the possibility of their elimination in mitochondria, a pronounced accumulation of H^+^ ions takes place. When the rate of oxygen uptake, cardiac output, and heart rate reach their maximum values, and the pH in the muscle decreases to extremely low values, leading to a decrease in muscle contractility, maximum oxygen consumption (VO_2_max) is recorded. Thus, VO_2_max is an integral indicator associated with the maximum performance of the oxygen transport system, because it determines the delivery of oxygen to all active tissues, not only to muscles. Therefore, changes in VO_2_max with enhanced training conditioning will not necessarily reflect changes in oxygen consumption by the main working muscles. It is worth noting that the relative loading intensity, expressed as a % of VO_2_max, may not reflect the actual physiological power of the exercise. The AT can vary significantly depending on age and level of physical fitness. In an untrained person, it averages 60% of the VO_2_max [161] and in trained athletes, it can reach 90% or more. 

On average, a person can maintain loads at the VO_2_max level for 3–8 min, depending on their training level [162]. The physiological mechanism for the formation of fatigue during physical work beyond the AT is likely associated with the accumulation of H^+^ protons [137], which may interfere with the release of calcium from the sarcoplasmic reticulum, reduce the sensitivity of troponin C to calcium [163], and also have an inhibitory effect on the activity of some enzymes of anaerobic metabolism (phosphofructokinase). In addition, acidosis has been reported to increase the number of non-productive actomyosin interactions and reduce the actin filament sliding velocity, increasing the time that myosin spends in an actin-bound state by slowing the rate of ADP release [164]. Thus, the amount of ATP that can be resynthesized during exercise performed beyond the AT will depend on the CrP content and the buffering capacity of the fiber.

During muscular work close to maximum anaerobic power, in the muscle groups bearing the main load, the maximum number of fibers available for recruitment is used, as well as all energy supply pathways associated with both anaerobic and aerobic resynthesis of ATP [165,166]. Nevertheless, the main source of energy supply for the actomyosin interaction in most working fibers is CrP, due to the high rate of phosphorylation of ADP by the creatine phosphokinase reaction. The rate of CrP consumption, determined by the ATPase activity of myosin, is at its maximum in fast glycolytic fibers. A decrease in the rate of creatine phosphokinase reaction, with reduced substrate availability, will significantly diminish the mechanical output of these fibers.

Thus, we see that the contribution of various factors determining muscle fatigue will obviously depend on the power range in which muscle fatigue testing is carried out. Despite the fact that functional unloading affects mostly type I fibers and low-intensity loads should be used (under which only type I muscle fibers are active) when studying fatigue under conditions of functional unloading, in most cases, the load on the muscle is in the zone above the anaerobic threshold (45% of the one-repetition maximum) and lasts from 1 to 5 min [9,12,14]. However, a number of experimental data indicate that the function of oxidative fibers contributes to fatigue during functional load and at high muscle power. In particular, in a series of experiments with the introduction of B-GPA (a creatine phosphokinase inhibitor that reduces the content of CrP in skeletal muscles during rat hindlimb suspension), fatigue tests were performed with ex vivo electrical stimulation of the soleus muscle. The initial contraction corresponded to the maximum force of contraction of this muscle, so the test was performed within a power range exceeding the AT. In this case, the contraction time was 1 min [91] or 5 min [167]. It could be assumed that a decrease in CrP levels would lead to an increase in muscle fatigue compared to the placebo group. However, in both studies, the opposite effect was observed. The introduction of B-GPA led to the prevention of muscle fatigue caused by functional unloading. This paradoxical effect can be associated with the positive effect of B-GPA on mitochondrial density—apparently, in this case, the improvement in the ability of oxidative fibres to utilize H^+^ due to preserved mitochondrial density was more significant than the reduced reserves of creatine phosphate.

Despite the lack of fatiguability tests within the different power ranges of muscle activity, the roles of several muscle metabolites were investigated under muscle unloading.

**Inorganic phosphate.** It has been shown that, in addition to creatine, inorganic phosphate formed during hydrolysis can impair myofibril function, reduce calcium release from the sarcoplasmic reticulum (SR), and therefore, contribute to a decrease in cross-bridge activation [168]. Pathare et al. [169] showed a significant inverse relationship between resting inorganic phosphate and specific strength. The work of Allen et al. [170] described some of the possible mechanisms of impaired calcium release from the sarcoplasmic reticulum, which is one of the causes of muscle fatigue: excessive accumulation of extracellular potassium after the action potential; decreased intracellular ATP; increased Mg^2+^ concentration. These reduce the efficiency of the calcium channel opening in the SR as well as decreasing the amount of Ca^2+^ available for release. This occurs due to the precipitation of Ca^2+^ by inorganic phosphate.

After 2 weeks of plaster immobilization of the gastrocnemius muscle, inorganic phosphate accumulated, and Pi/CrP levels increased [171], while pH, lactate, and ATP levels did not change in this experiment compared to the control. Moreover, under 10 days of rat hind limb suspension, a decrease in the level of Pi/CrP [172] and an increase in the level of CrP was found in the soleus and gastrocnemius muscles compared to the control group. After 2 weeks of suspension, increased lactate and glycogen levels were found in the soleus muscle [173]. An 18-day restriction of mobility in primates (staying in an orbital module under terrestrial conditions) led to the accumulation of lactate in the fast fibers of the gastrocnemius but not the soleus [174]. It is possible that the accumulation of lactate under muscle unloading may be associated either with a decrease in mitochondrial functions or with a decrease in capillarization which leads to a reduced rate of lactate removal from muscle fibers.

**ROS.** It is known that reactive oxygen species (ROS) generated during mitochondrial respiration can contribute to fatigue by oxidizing cellular proteins such as the sodium-potassium pump, myofilaments, dihydropyridine, and ryanodine channels. This results in decreased calcium release from SR and decreased sensitivity of actomyosin complexes to calcium [175]. Under rodent hindlimb suspension and denervation, the accumulation of reactive oxygen species has been shown after the first six hours of intervention [50,176]. The accumulation of ROS has also been shown under conditions of muscle immobilization [177]. Activation of signaling pathways associated with an increase in ROS was shown in a proteomic analysis of the slow muscle fibers muscles of astronauts after a 6-month space flight [178]. Many studies have been devoted to the reasons for the accumulation of ROS during functional unloading; however, they still remain unclear. It is known that mitochondria contribute to the accumulation of ROS at different times and under different models of functional unloading [177,179] leading to the accumulation of angiotensin II which causes activation of NADPH oxidases NOX2 [180] and accumulation of myoplasmic calcium [81].

**Glycogen.** Glycogen content was shown to influence SR calcium release in highly trained athletes [181]. At the same time, glycogen is a source of energy for muscle and a negative regulator of AMPK activity [182], so both glycogen accumulation and glycogen depletion could affect muscle fatigue resistance. There is a limited number of studies dedicated to glycogen content under disuse conditions, and usually they show its accumulation. The increase in glycogen content was shown in the soleus muscle during hind limb unloading of different durations—from 12 h for as long as 14 days. After 14-day hind limb suspension, the slow-type fibers of suspended animals utilized more glycogen than those of control animals during muscle contraction [52,173]. Both 6-day and 60-day bed rest led to glycogen accumulation in the vastus lateralis muscle [183]. 6-day dry immersion also led to glycogen accumulation in the soleus muscle [87]. In contrast, two weeks of cast immobilization caused glycogen depletion in the vastus lateralis muscle [184]. The effect of glycogen content alterations under disuse remains unclear, as it should depend of the direction of its change as well as on the method of fatigue assessment. 

**Macroergic phosphates.** The soleus muscle is characterized by a complete cessation of contractile activity when the supporting afferentation is eliminated (i.e., at the moment of loss of the supporting stimulus). Cessation continues at least during the first day of functional unloading, and then is replaced by a gradual restoration of activity to the control level (by day 14)—the so-called “spontaneous tonic muscle activity” [185]. At the initial stage of unloading, while mitochondrial density is still preserved, the cessation of muscle contractile activity is accompanied by the accumulation of ATP [55,167] and, accordingly, a decrease in AMP content. This leads to a decrease in the level of phosphorylation of AMPK (AMP-dependent protein kinase, the activator of mitochondrial biogenesis) and the expression of the slow, fatigue-resistant isoform of myosin-heavy chains. A decrease in the level of AMPK phosphorylation was shown already on the 3rd day of “dry” immersion, as well as after 12, 24 h, and 3 days of rat hind limb suspension [42,49,53,55,118,186]. It has also been shown that on the 14th day of unloading, the ATP content decreases [55], which may be due to its consumption while spontaneous tonic activity or due to a decrease in mitochondrial density in the later stages of unloading. Under conditions of muscle unloading, it was shown that on day 1, the level of AMPK phosphorylation was significantly reduced relative to the control; on day 3, it showed a downward trend; on day 7 it was equal to the control level [118]; on day 14, there was a significant increase in phosphorylation [187], which corresponds to the dynamics of ATP changes. Our laboratory has shown that preventing the accumulation of ATP after the first day of suspension by administering the creatine kinase blocker beta-guanidine propionic acid prevents a decrease in the level of AMPK phosphorylation and a decrease in the expression of mRNA of a number of regulators of mitochondrial biogenesis [54]. Administration of beta-guanidine propionic acid during a week of suspension prevents increased fatigue in the soleus muscle of rats, as well as the transformation of the myosin phenotype to the “fast” side and a decrease in the number of mitochondrial DNA copies [91]. Separately, it is worth noting that the administration of beta-guanidine propionic acid against the background of 10 days of suspension, when AMPK activity is already equal to or exceeds control values, also has a positive effect on muscle fatigue during suspension [188].

## 7. Conclusions

Skeletal muscle unloading leads to increased muscle fatigue, primarily in the muscles of the lower limbs. The unloading-induced changes leading to increased fatigue include both neurobiological processes (decreased corticospinal excitability, changes in intrinsic motor neuron activity) and intramuscular processes. The development of intramuscular fatigue is contributed by the transformation of soleus muscle fibers from a fatigue-resistant, “oxidative” “slow” phenotype to a “fast” “glycolytic“ one. In turn, this transformation is triggered by the inactivation of muscle fibers in the early stages of muscle unloading, the accumulation of high-energy phosphates and calcium ions in the myoplasm, as well as a decrease in NO. These secondary messengers trigger signaling processes leading to structural changes in muscles that, in turn, cause increased fatigue.
